# Genotyping of high-risk anal human papillomavirus (HPV): ion torrent-next generation sequencing vs. linear array

**DOI:** 10.1186/s12985-017-0771-z

**Published:** 2017-06-13

**Authors:** Rebecca G. Nowak, Nicholas P. Ambulos, Lisa M. Schumaker, Trevor J. Mathias, Ruth A. White, Jennifer Troyer, David Wells, Manhattan E. Charurat, Søren M. Bentzen, Kevin J. Cullen

**Affiliations:** 1 Institute of Human Virology, University of Maryland School of Medicine, Baltimore, MD USA; 2 University of Maryland Marlene and Stewart Greenebaum Cancer Center, University of Maryland School of Medicine, 22 S. Green St. N9E17, Baltimore, MD 21201 USA; 3 Department of Med Hematology and Oncology, Columbia University, New York, NY USA; 4 National Human Genome Research Institute, Rockville, MD USA; 5 Cancer Research Technology Program, Frederick National Laboratory for Cancer Research, Frederick, MD USA

**Keywords:** Human papillomavirus, Multiple HPVs, Next-generation sequencing, Ion-Torrent, Linear Array

## Abstract

**Background:**

Our next generation sequencing (NGS)-based human papillomavirus (HPV) genotyping assay showed a high degree of concordance with the Roche Linear Array (LA) with as little as 1.25 ng formalin-fixed paraffin-embedded-derived genomic DNA in head and neck and cervical cancer samples. This sensitive genotyping assay uses barcoded HPV PCR broad-spectrum general primers 5+/6+ (BSGP)5+/6+ applicable to population studies, but it’s diagnostic performance has not been tested in cases with multiple concurrent HPV infections.

**Methods:**

We conducted a cross-sectional study to compare the positive and negative predictive value (PPV and NPV), sensitivity and specificity of the NGS assay to detect HPV genotype infections as compared to the LA. DNA was previously extracted from ten anal swab samples from men who have sex with men in Nigeria enrolled on the TRUST/RV368 cohort study. Two-sample tests of proportions were used to examine differences in the diagnostic performance of the NGS assay to detect high vs. low-risk HPV type-specific infections.

**Results:**

In total there were 94 type-specific infections detected in 10 samples with a median of 9.5, range (9 to 10) per sample. Using the LA as the gold standard, 84.4% (95% CI: 75.2–91.2) of the same anal type-specific infections detected on the NGS assay had been detected by LA. The PPV and sensitivity differed significantly for high risk (PPV: 90%, 95% CI: 79.5–96.2; sensitivity: 93.1%, 95% CI: 83.3–98.1) as compared to low risk HPV (PPV: 73%, 95% CI: 54.1–87.7; sensitivity: 61.1, 95% CI: 43.5–76.9) (all *p* < 0.05). The NPV for all types was 92.5% (95% CI: 88.4–95.4). The NPV and specificity were similar for high and low risk HPVs (all p > 0.05). The NGS assay detected 10 HPV genotypes that were not among the 37 genotypes found on LA (30, 32, 43, 44, 74, 86, 87, 90, 91, 114).

**Conclusions:**

The NGS assay accurately detects multiple HPV infections in individual clinical specimens with limited sample volume and has extended coverage compared to LA.

**Electronic supplementary material:**

The online version of this article (doi:10.1186/s12985-017-0771-z) contains supplementary material, which is available to authorized users.

## Background

Identifying type-specific infections of human papillomavirus (HPV) is fundamental for understanding carcinogenic risk in epidemiologic studies, particularly in high burden areas such as sub-Saharan Africa. We recently developed a next-generation sequencing (NGS)-based HPV genotyping assay that shows 92% concordance on type-specific infections with the Roche Linear Array (LA) in cases with head and neck or cervical cancer [1]. The assay uses barcoded HPV PCR broad-spectrum general primers 5+/6+ (BSGP5+/6+) [1] that have a high sensitivity for HPV type specific infections and for multiple HPV infections as compared to earlier versions of the primer set (GP5+/6+) [2]. Prior studies evaluating the benefits of next generation sequencing for the detection of HPV have incorporated different primer sets [3–7] or used whole genome sequencing [8–10] for viral discovery and have not evaluated the effectiveness of the BSGP5+/6+ primer set in a next generation sequencing assay as compared to the PGMY09/11 primer set used in LA. Some studies have shown that these broad-spectrum primer-based PCR methods may be less sensitive at detecting multiple infections [11, 12]. Our prior study could not test the performance of the NGS assay in samples with multiple infections because the head and neck squamous cell carcinomas and cervical cancer specimens tested were primarily associated with a single HPV type-specific infection, with the exception of one sample that had two type-specific infections. Therefore, the objective of this study was to estimate the diagnostic performance of a NGS assay that employs BSGP5+/6+ primers as compared to the LA to detect a high number of concurrent HPV type-specific infections in anal swab samples collected from an epidemiologic study of men who have sex with men.

## Methods

### Samples

Genomic DNA was previously extracted from anal swab samples and genotyped on LA to determine the prevalence of HPV type-specific infections between HIV-positive and HIV-negative men who have sex with men in Abuja, Nigeria [13]. In brief, DNA was extracted from 250 μL of Aptima Specimen Transport medium (Hologic, San Diego, CA) using the QIAmp MinElute Media Kit (Qiagen, Valencia, CA). DNA was resuspended in 100 μL of Buffer AVE, quantified by NanoDrop (ng/μL) and stored at -20^0^ C. A 10-μL aliquot of the purified DNA was amplified using the PGMY 09/11 L1 consensus primer system which co-amplifies 37 HPV genotypes and a human β-globin internal control target. Both high- and low-risk HPV genotypes were detected using the LA (Roche Diagnostics, Indianapolis IN). High-risk HPV included 13 type specific infections: 16, 18, 31, 35, 39, 45, 51, 52, 56, 58, 59, and 68 [14]. For any HPV (both low and high-risk), the median number of infections was 4, (interquartile range [IQR]: 2–8, range: 0–15); 38% (59/154) of the specimens had 2–5 infections and 38% (58/154) had 6–15 infections. Approximately 49% (76/154) of the specimens had two or more high-risk HPV infections (median: 1, IQR:0–3, range 0–7).

### Laboratory

For the present study, 10 samples of genomic DNA positive for multiple high-risk HPV infections were selected. Quantified genomic DNA (20 ng) were included in HPV library amplification as previously described [1]. In brief, all samples were included in the sequencing pool at a standardized concentration of ~500pM, as determined by the BioAnalyzer. Samples without library product detection were included in the pool at equal volumes. Pooled samples were purified and then quantified for emulsion template preparation on the Qubit 2.0 Fluorometer and prepared using Ion personal genome machine (PGM) 200 kits on the OneTouch 2. Sequencing was performed on the Ion Torrent PGM using the 200 v2 sequencing chemistry and 316v2 chips.

Data processing was performed by the ion torrent server, using Torrent Suite v4.4.3, and mapped to the full genomic sequences of HPV downloaded from the Papillomavirus Episteme (PaVE) database with a minimum score of AQ17. Further filtering of only reads >100 bp was performed using NGSUtils. A sample contained >5000 reads to be included in the analysis and the reads for each type specific infection accounted for more than 0.05% of the total number of reads to be called positive.

### Statistical analyses

The median and interquartile range of the number of genotypes identified per sample were estimated for any HPV infection, high-risk HPV, and low-risk HPV infection. To compare the NGS-HPV genotyping assay with LA, the presence or absence of 33 type-specific infections that were detectable by both assays were included in the analysis (6, 11, **16**, **18**, 26, **31**, **33**, **35**, **39**, 40, 42, **45**, **51**, 53, 54, **56**, **58**, **59**, 61, 62, 66, 67, **68**, 69, 70, 71, 72, 73, 81, 82, 83, 84, 89. Bolding indicates high-risk). There were 4 type-specific infections (52, 55, 64, and IS39 (82 subtype) that were not included in the comparison. In the LA assay, HPV 52 cross reacts with 35 and 58. If either 35 or 58 are present, then 52 may be underdiagnosed in the LA assay. For the other three genotypes, genomic sequences were not present in the PaVE database and were not available for mapping and therefore could possibly be underdiagnosed in the NGS assay. In total there were 330 type-specific infections that could be detected by both assays.

To estimate the ability of the NGS assay to detect the same types of infections detected on LA, we calculated the positive predictive value [(true positive/(true positive + false positive))*100] and the negative predictive value [(true negative/(true negative + false negative))*100] for any HPV, and separately for high-risk HPV and low-risk HPV with the associated 95% confidence intervals. To estimate the ability of the NGS assay to correctly identify those with and without HPV genotypes, we calculated the sensitivity [(true positive/(true positive + false negative))*100] and the specificity [(true negative/(true negative + false positive))*100] for any HPV, and separately for high-risk HPV and low-risk HPV with the associated 95% confidence intervals. Two-sample tests of proportions were used to compare the performance of the NGS-HPV assay for high and low risk HPV infections relative to the LA. Analyses were performed using Stata Statistical Software: Release 13 (College Station, TX: Stata Corp LP).

## Results

**Fig. 1:**
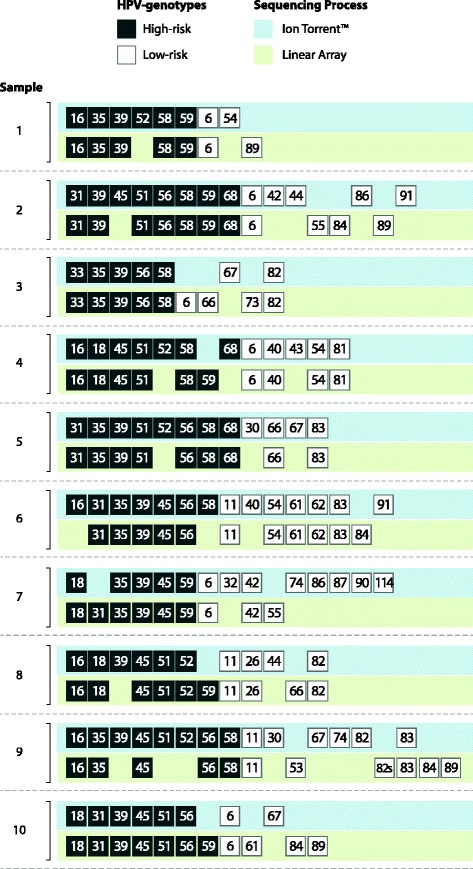
High and low-risk HPV type-specific infections detected by NGS and LA (*n* = 10)

The median number of HPV type-specific infections in the 10 samples as detected by LA was 9.5 (IQR: 9–10), the median number of high risk HPV was 5 (IQR: 5–7), and the median number of low risk HPV was 4 (IQR: 2–4) (Fig. 1). In total, the NGS assay detected 76 of the 94 type-specific infections detected by LA. Using the LA as the gold standard, 84.4% (95% CI: 75.2–91.2) of the same HPV type-specific infections detected on the NGS assay had been detected on the LA (Table 1). The NGS HPV assay was more likely to detect the same anal high risk HPV (PPV: 90.0%, 95% CI: 79.5–96.2) as compared to the low risk HPV (PPV:73.3%, 95% CI: 54.1–87.7) (*p* = 0.04). The sensitivity of the NGS assay was higher for high risk HPV as compared to low risk infections (*p* = 0.0001). Interestingly, the NGS HPV assay never detected any of the low risk HPV types 84 and 89. If we exclude these two types, the sensitivity of the NGS assay becomes 78.6%, 95% CI: 59.0–91.7. The NPV for all types was 92.5% (95% CI: 88.4–95.4) and the NPV and specificity were similar for low and high risk HPVs (*p* = 0.80, *p* = 0.16, respectively). Overall, the NGS HPV assay detected an additional 10 HPV genotypes that were not among the 37 genotypes found on the LA (30, 32, 43, 44, 74, 86, 87, 90, 91, 114) (Fig. 1).

**Table 1:** Positive and negative predictive value, sensitivity and specificity of NGS-HPV vs Linear Array

	%PPV (95% CI)	%NPV (95% CI)	Sensitivity (95% CI)	Specificity (95% CI)
HR-HPV	90.0 (79.5–96.2)	93.3 (83.8–98.2)	93.1 (83.3–98.1)	90.3 (80.1–96.4)
LR-HPV	73.3 (54.1–87.7)	92.2 (87.3–95.7)	61.1 (43.5–76.9)	95.4 (91.1–98.0)
All HPV	84.4 (75.2–91.2)	92.5 (88.4–95.4)	80.9 (71.4–88.2)	94.1 (90.2–96.7)

*Abbreviations*: *NGS* Next generation sequencing, *HR-HPV* High risk human papillomavirus, *LR-HPV* Low-risk human papillomavirus, *PPV* Positive predictive value, *NPV* Negative predictive value, *CI* Confidence intervals

## Discussion

Our study confirms that the NGS assay was able to detect multiple HPV infections, particularly the high risk type-specific infections. The NGS assay detected 90% of the same high risk HPV as the LA and its sensitivity or true positive rate for high-risk HPV was 93%. More specifically, the NGS assay can detect HPV-52, a high risk HPV that is underdiagnosed on the LA because it cross reacts with other type-specific infections. For the true negative infections, the NGS assay was able to detect 93% of the same types not detected on the LA. The NPV and the specificity were similar regardless if they were low or high risk infections. These findings are consistent with an earlier study that found that the additional eight forward broad-spectrum primers and two backward primers significantly improved the ability of this primer set to detect multiple infections [2].

The Ion Torrent NGS assay may not be as sensitive for detecting all HPV type-specific infections as the new multiplex type-specific HPV E7 PCR system [11, 12], but its purpose is to detect the known oncogenic type-specific infections from small amounts of DNA for multiple samples in longitudinal studies. The slightly lower positive predictive value for all types of HPV was in part driven by the low PPV for low risk infections. For epidemiologic studies focused on high risk infections, the NGS assay is a suitable alternative to the LA. For studies that have valuable archived samples, it is particularly advantageous as a genotyping assay because it uses as little as 20 ng of genomic DNA. For men, the prevalence of high risk HPV remains high and does not significantly decline with age as seen in women [15, 16]. A better understanding of gender differences in the natural history of HPV could be facilitated by diagnostics that require small sample volumes.

There is a possible limitation to our study. We oversampled specimens that had a large number of high-risk HPV infections which may have reduced the number of low-risk types included in the total sample. This may in turn have affected our estimates of the positive predictive value and sensitivity of the assay to detect low-risk HPV infections. Still, in larger studies, the capability to detect multiple oncogenic type-specific infections is more relevant to understanding risk factors for progression to cancer.

## Conclusion

Despite this limitation, our study demonstrates that the NGS assay is comparable to the LA in detecting multiple high-risk HPV infections. The NGS assay could provide a diagnostic tool for large epidemiologic studies evaluating the natural history of HPV and identifying those most at risk of developing HPV-associated malignancies. The reagent and material cost per sample is significantly lower with the NGS assay, although that will have to be balanced against the capital investment in the Ion Torrent platform.

## Additional file

Additional file 1: The tabular data used in the analyses for this manuscript is included as an excel file. This data includes both the LA and NGS results for each of the types detected on the 10 samples. (XLS 57 kb)

## Abbreviations

BSGP: Broad-spectrum general primers
HPV: Human papillomavirus
LA: Roche Linear Array
NGS: Next generation sequencing
NPV: Negative predictive value
PaVE: Papillomavirus Episteme
PGM: Personal genome machine
PPV: Positive predictive value
